# A systematic review of behavioural and exercise interventions for the prevention and management of chemotherapy-induced peripheral neuropathy symptoms

**DOI:** 10.1007/s11764-021-00997-w

**Published:** 2021-03-12

**Authors:** Mary Anne Lagmay Tanay, Jo Armes, Rona Moss-Morris, Anne Marie Rafferty, Glenn Robert

**Affiliations:** 1 Florence Nightingale Faculty of Nursing, Midwifery and Palliative Care, King’s College London, London, UK; 2 School of Health Sciences, Faculty of Health and Medical Sciences, University of Surrey, Guildford, UK; 3 Institute of Psychiatry, Psychology and Neuroscience, King’s College London, London, UK

**Keywords:** Systematic review, Chemotherapy-induced peripheral neuropathy, Cancer, Cancer survivorship, Behavioural intervention, Exercise

## Abstract

**Background:**

Chemotherapy-induced peripheral neuropathy (CIPN) can result in functional difficulties. Pharmacological interventions used to prevent CIPN either show low efficacy or lack evidence to support their use and to date, duloxetine remains the only recommended treatment for painful CIPN. Non-pharmacological interventions such as exercise and behavioural interventions for CIPN exist.

**Purpose:**

The aims were to (1) identify and appraise evidence on existing behavioural and exercise interventions focussed on preventing or managing CIPN symptoms, (2) describe psychological mechanisms of action by which interventions influenced CIPN symptoms, (3) determine the underpinning conceptual models that describe how an intervention may create behaviour change, (4) identify treatment components of each intervention and contextual factors, (5) determine the nature and extent of patient and clinician involvement in developing existing interventions and (6) summarise the relative efficacy or effectiveness of interventions to lessen CIPN symptoms and to improve quality of life, balance and muscle strength.

**Methods:**

A systematic search of Ovid Medline, Cochrane Library, EMBASE, PsycINFO, Health Management Information Consortium, Global Health and CINAHL was performed to identify articles published between January 2000 to May 2020, followed by OpenGrey search and hand-searching of relevant journals. Studies that explored behavioural and/or exercise interventions designed to prevent or improve symptoms of CIPN in adults who had received or were receiving neurotoxic chemotherapy for any type of cancer, irrespective of when delivered within the cancer pathway were included.

**Results:**

Nineteen randomised controlled trials and quasi-experimental studies which explored behavioural (*n*=6) and exercise (*n*=13) interventions were included. Four studies were rated as methodologically strong, ten were moderate and five were weak. Ten exercise and two behavioural interventions, including those that improved CIPN knowledge and self-management resources and facilitated symptom self-reporting, led to reduced CIPN symptoms during and/or after chemotherapy treatment.

**Conclusions:**

The extent of potential benefits from the interventions was difficult to judge, due to study limitations. Future interventions should incorporate a clear theoretical framework and involve patients and clinicians in the development process.

**Implications for Cancer Survivors:**

Our findings show exercise interventions have beneficial effects on CIPN symptoms although higher quality research is warranted. Behavioural interventions that increase patient’s CIPN knowledge, improve self-management capacity and enable timely access to symptom management led to reduced CIPN symptoms.

**Supplementary Information:**

The online version contains supplementary material available at 10.1007/s11764-021-00997-w.

## Introduction

Chemotherapy remains one of the main cancer treatments despite many side effects caused by damage affecting normal cell growth and function. Some chemotherapy drugs such as taxanes, platinum-based drugs and bortezomib cause injury and damage to the nerves causing peripheral neurological symptoms known as chemotherapy-induced peripheral neuropathy (CIPN) [1]. Felt mainly on the hands, feet or both, CIPN presents as a solitary or combination of symptoms such as numbness, tingling sensations, sharp pain, lack of temperature sensation and muscle weakness [2]. Other symptoms include hearing loss and tinnitus [3]. If not mitigated or managed appropriately, CIPN symptoms result in functional difficulties affecting day-to-day social, domestic and work activities [4, 5]. Some patients experience movement, balance and coordination problems and become more prone to injury and falls [6, 7]. A recent review of pharmacological interventions used to prevent CIPN either showed low efficacy or lacked evidence to support their use and to date, duloxetine remains the only recommended treatment for painful CIPN [8, 9]. Alternatively, studies and systematic reviews of non-pharmacological interventions such as exercise and behavioural interventions suggest these treatment approaches may have potential beneficial effects on reducing CIPN symptoms and may be appealing because patients do not have to take another drug to treat another symptom [10–13]. There are currently no recommended non-pharmacological treatments for CIPN [9]; a focus on addressing the psychological mechanisms influencing CIPN development is lacking.

Applying behavioural approaches may be useful because CIPN is associated with a wide range of psychosocial and secondary factors that contribute to patients’ experience of CIPN symptoms, such as poor or disturbed sleep, cancer or treatment-related anxiety and unhelpful cognitive behavioural responses to CIPN symptoms, such as underreporting of symptoms to get maximum dose or acceptance that CIPN symptom severity is equivalent to treatment efficacy [4, 14]. Behavioural interventions aim to influence and target behaviours, cognitions and/or emotions which perpetuate or worsen symptoms [15, 16]; they have been used to improve management of cancer disease symptoms and chemotherapy side effects such as fatigue and cognitive dysfunction [17–19]. These interventions usually include a range of components which aim to improve knowledge, encourage people to change their behaviour and/or the way they think about or emotionally respond to their symptoms. For example, behavioural interventions which target reductions in the severity or impact of symptoms have also been associated with improved physical health and coping skills [20, 21]. Engaging with useful behavioural responses by staff and patients can potentially aid early identification, assessment and mitigation of CIPN symptoms [4]. Similarly, exercise is recommended to improve symptoms of other forms of nerve damage such as diabetic neuropathy [22] and to optimise postural balance among older patients [23, 24]. Research also suggests that maintaining or increasing physical activity has beneficial effects on patients’ quality of life and physical functioning as well as improving ongoing treatment-related symptoms such as fatigue and CIPN [25].

To date, no review of CIPN-specific behavioural interventions has been conducted. A systematic review, focused on studies involving patient self-management to reduce symptoms of peripheral neuropathy (PN) caused by a range of conditions such as diabetes, HIV and other autoimmune disorders, reported that self-initiated interventions may reduce self-reported PN symptoms [26]. But results may not be wholly applicable when managing neuropathy in the context of cancer chemotherapy because CIPN is an unintended consequence of chemotherapy treatment that also brings associated psychosocial complexities [4]. Greater understanding of specific components and how available interventions work, or do not work, in different contexts can support the development and successful implementation of future interventions to encourage helpful behavioural responses to CIPN symptoms among patients with symptoms or those who are about to receive neurotoxic chemotherapy. Two systematic reviews of exercise in CIPN exist [10, 12]. Both reviews described available exercises; the more recent review included a summary of intervention components [12]. Despite including a heterogeneous group of exercise studies and interventions, both reviews indicated that exercise interventions show promise in preventing and mitigating CIPN symptoms. However, it is unclear which exercise interventions showed the greatest benefit as analyses of contextual factors, intervention components, conceptual underpinnings and mechanisms of action were not elaborated.

To understand which behavioural and exercise interventions are safe and effective, it is vital to identify the core intervention components and how these are best delivered [27, 28]. Similarities and differences in underpinning conceptual models and mechanisms of actions of interventions [29] should be considered to understand relative intervention effectiveness [30]. The Medical Research Council (MRC) [29] recommends careful consideration of underpinning conceptual or theoretical models of complex interventions. Several studies employed a theory-based approach in the development of interventions within cancer care such as the use of behaviour change theories. For example, Bradbury et al. [31] developed a digital intervention to improve quality of life in cancer survivors guided by the Behaviour Change Wheel [32] and Normalisation Process Theory [33]. Likewise, a study by Corbett et al. [34] integrated the use of self-regulation model [35] to describe fatigue after cancer, and the Behaviour Change Technique (BCT) Taxonomy v.1 [36] to describe components of the intervention. To enable clarity when making intervention assumptions, the MRC recommends the use of logic models to visually present the core components of the intervention, how they interact to produce change, the anticipated outcomes, and resources and structures in place to ensure implementation [29].

Previous research shows that patients felt inadequately prepared for CIPN by the healthcare team before commencing chemotherapy which consequently affected early recognition and management of CIPN symptoms [4, 5]. However, there is also evidence that patients are not reporting their symptoms, fearing their chemotherapy dose might be reduced or stopped [4]. The MRC highlights how engaging stakeholders (e.g. clinicians, patients and their carers) can improve the likelihood that interventions which are relevant and effective are adopted into routine practice [29]. Such involvement of stakeholders—through approaches such as co-production and co-design—can therefore help improve patient experience and illness burden, treatment and economic costs [37]. Robert et al. [38] argue that healthcare services and interventions are traditionally shaped by metrics which lack active participation of patients and their carers in identifying needs, determining priorities and implementing change. Studies have shown that patients are able to translate their experiences into improvement priorities that should be considered when developing patient-centred cancer services [39] and appropriate information for service users [40]. When patients and clinicians work together over a period and throughout the change process, shared decisions and patient-centred services ensue but more importantly, a synergistic effect of ‘user-centred design, technological innovation and human learning’ is enriched [37 , p. 2].

The current review systematically identified and appraised evidence relating to existing behavioural and exercise interventions focussed on preventing or managing symptoms of CIPN. The specific objectives were to:

- describe psychological mechanisms of action by which an intervention influenced CIPN symptoms;
- determine the underpinning conceptual models that describe how an intervention may create behaviour change;
- identify the treatment components of each intervention and contextual factors;
- determine the nature and extent of patient and healthcare professional involvement in developing existing behavioural and exercise interventions; and
- summarise the relative efficacy or effectiveness of interventions to lessen neuropathic pain and CIPN symptoms and to improve quality of life, balance and muscle strength.

## Methodology

The review was guided by the Cochrane Handbook for Systematic Reviews of Interventions [41], Guidance on the Conduct of Narrative Synthesis in Systematic Reviews [42] and Preferred Reporting Items for Systematic Reviews and Meta-Analysis (PRISMA) guidance [43].

### Search strategy

A systematic online search of studies published from January 2000 to the 20th of May 2020 was conducted using the following databases: Ovid Medline, Cochrane Library, EMBASE, PsycINFO, Health Management Information Consortium, Global Health and CINAHL. These databases support systematic searching of wide range of topics in health and healthcare. An exemplar of the search protocol is presented in Online Resource S-1. Additional manual searching of included studies was carried out by screening reference lists of included studies and hand-searching relevant journals until the 20th of May 2020 (Journal of Peripheral Nervous System, Supportive Care in Cancer, Psycho-Oncology and Journal of Clinical Oncology). OpenGrey was also searched after completing database and manual searches to identify unpublished work relevant to the research question.

### Inclusion and exclusion criteria

We included randomised controlled trials or quasi-experimental studies published in peer-reviewed journals that explored behavioural and/or exercise interventions delivered by health providers and designed to prevent or improve symptoms of CIPN in adults who had received or were receiving neurotoxic chemotherapy for any type of cancer, irrespective of when they were delivered in the cancer pathway. Behavioural interventions (BIs) focused on changing behaviour, cognition, attitudes and/or emotions [44]. Exercise interventions (EIs) included types of physical activity consisting of planned, structured and repetitive bodily movement done to improve and/or maintain one or more components of physical fitness [45, 46].

Studies were excluded if they evaluated interventions for other types of neuropathy such as those due to diabetes, trauma, nutritional deficiency, infections or vascular problems because their aetiologies are different from CIPN. Studies that tested pharmacological interventions, dietary treatment or nutritional supplementation and complementary and alternative medicine as defined in the National Health Service website [47] were outside the scope of this review. The review was also limited to studies involving adult patients and published in the English language.

### Study selection

MT performed the literature search, and scanned all articles by title and abstract. MT and JA independently screened articles in full text for eligibility. This was followed by discussion with the co-authors (GR, RMM and AMR) to establish consensus on which studies were included, particularly when there was ambiguity.

### Quality appraisal

Criteria set out in the Effective Public Health Practice Project (EPHPP) quality assessment tool [48] allows methodological quality assessment of studies which evaluated intervention effectiveness using a range of quantitative methodologies [49]. Six areas of study quality were assessed, namely selection bias, study design, confounders (age, health status, drug type and dose), blinding process, data collection methods and reasons for dropouts or withdrawals. Methodological quality assessment was independently carried out by MT and JA and then verified by GR.

### Data extraction

A data extraction tool based on Cochrane Handbook Recommendations [41] was developed to extract research data pertaining to study design, setting, number and demographic profile of participants, methods, outcomes, measurement tools and timing of assessments. Characteristics and details of the interventions were extracted using a tool based on the Template for Intervention Description and Replication (TIDieR) Tool [28] which included treatment components, materials used, processes/procedures, who was involved in delivering training, how the intervention was individualised or modified, acceptability and contextual factors. For studies that involved secondary data analysis, key intervention components were extracted from either the primary article or an earlier published article of the intervention. The content of each intervention was mapped onto the Behavioural Change Taxonomy v.1 to detail and categorise the behavioural change techniques (BCT), which are potentially active ingredients of the interventions [36]. MT conducted the data extraction; consensus was achieved through discussion among authors. Listed below are descriptions of the BCTs [36] which will be specifically discussed later in this paper.

- Action planning: prompt detailed planning of performance of the behaviour including at least one of context, frequency, duration and intensity.
- Instruction on how to perform a behaviour: advise or agree on how to perform a behaviour.
- Habit formation: prompt rehearsal and repetition of the behaviour in the same context repeatedly so that the context elicits the behaviour.
- Giving prompts and cues: introduce or define environmental or social stimulus with the purpose of prompting or cueing behaviour. The prompt or cue normally occurs at the time or place of performance.
- Goal setting (behaviour) BCT: when there is a set or agreed terms of the behaviour to be achieved.

### Methods of analysis

Data synthesis comprised two parts namely, narrative analysis and intervention synthesis.

Firstly, a narrative synthesis was conducted according to Guidance on the Conduct of Narrative Synthesis in Systematic Review *[*42*].* To determine the characteristics of the included studies, extracted data were initially synthesised using textual descriptions. These were subsequently grouped, clustered and presented in tabular form. Contextual factors, study design and settings, participant characteristics, outcomes and outcome measures were examined to explore the relationships within and between the studies.

Intervention synthesis for interventions with similar features and functions was grouped together into two broad sub-categories: (1) behavioural and (2) exercise interventions. Intervention synthesis was guided by the TIDieR checklist [28]. Following the process of Common Components Hybrid method, syntheses of interventions involved listing of all components, coding and selecting common components within and across interventions [27].

Guided by the MRC Guidance on Process Evaluation of Complex Interventions [29], a diagrammatic summary of evidence to date was developed to illustrate the context, underpinning conceptual models, intervention components, psychological mechanisms of action and their effect on intended outcomes.

## Results

### Study selection

**Fig. 1:**
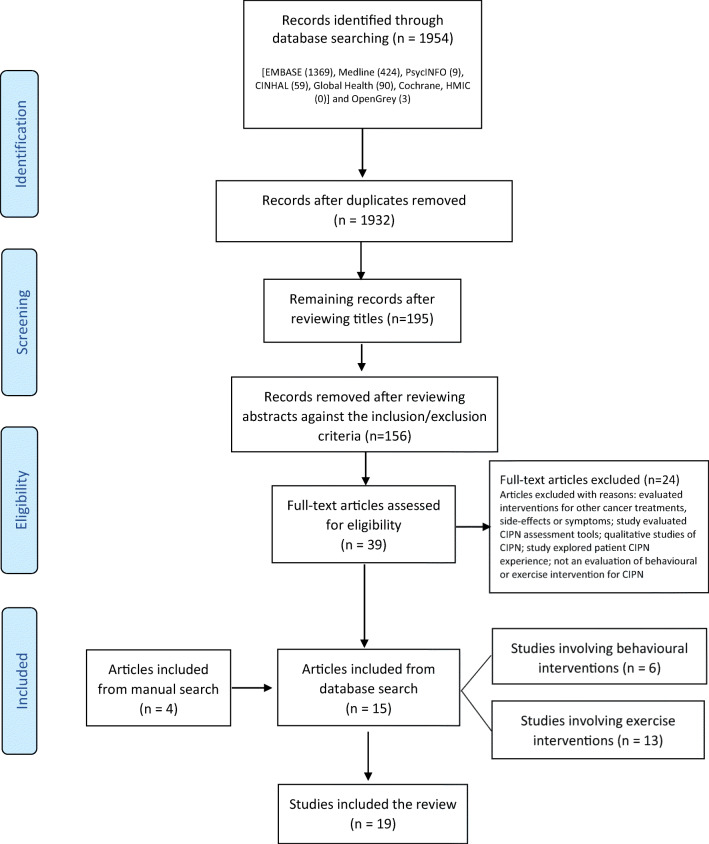
PRISMA flowchart of study selection

The search strategy generated 1954 articles. After removing duplicates, reviewing titles and abstracts, 39 articles were read in full. Twenty-four studies were excluded for reasons listed in Fig. 1. Fifteen studies from the databases and a further four studies through manual searching were identified as meeting the inclusion criteria. No studies were identified through Open Grey search. In total, 19 studies were included in the review involving 1,538 patients who had CIPN or were at high risk of developing CIPN. Six studies evaluated seven behavioural interventions (BIs) and 13 evaluated exercise interventions (EIs). The study selection process and results are illustrated in Fig. 1.

### Quality appraisal

Overall, four of the 19 studies were rated as methodologically strong, ten as moderate and five as weak (see Online Material S-2). Earlier studies which explored exercise interventions were noted to be weak; more recent studies were mostly methodologically moderate. Regarding patient selection bias, only two studies were rated weak, while the remainder were rated mainly moderate (*n*=13) or strong (*n*=4). Confounders were controlled for most studies (*n*=13). For blinding, only one study was ranked strong. In the remaining studies, either the blinding process was not explained, outcome assessors were aware of the exposure status of the participant or participants were aware of the research question. Reliable and valid outcome measures were used in most studies. Regarding dropouts, seven studies had a rate of less than 20%, and were, therefore, rated as strong.

The quality of reporting of interventions was variable when assessed against the TIDieR checklist [28]. Three BI studies reported adherence in percentages but only one [13] elaborated on factors which affected adherence. Planned and actual assessment of fidelity were reported only by one EI study [50]. All remaining studies either did not report or insufficiently reported details about fidelity and adherence.

### Study characteristics

All studies included in this review were approved by ethics committees and obtained consent from study participants. A summary of characteristics of all included studies is presented in Table 1.

**Table 1:** Summary of characteristics of included studies involving interventions

Study	Study design	Intervention (I) vs control/comparator (C)	Stage of treatment	Participants characteristics	Outcomes (and measurement tools)
Behavioural interventions					
Given *et al.* (2008) [51] USA	Randomised controlled trial (sub-analysis)	**I:** Automated Telephone Symptom Management (ATSM) Automated system made one call to the patient to assess 15 various symptoms on weeks 1, 2, 3, 4, 6 and 8 during treatment. Up to 4 symptoms above severity threshold were managed according to guide and were reviewed on the next call. **C:** Nurse-Administered Symptom Management (NASM) Nurse practitioner made one call to the patient to assess 15 various symptoms on weeks 1, 2, 3, 4, 6 and 8 during treatment. Up to 4 symptoms above severity threshold were managed according to guide and were reviewed on the next call.	During chemotherapy treatment	174 female participants with breast cancer, mainly Caucasians (87%) who received unspecified neurotoxic drugs	**Primary outcomes:** Total symptom severity score summed across multiple symptoms (scale 1–10) Time to response (number of days between the contact date of severe symptom and date of sustained response. Total number of symptoms reaching threshold
Tofthagen *et al.* (2016) [52] USA	Single-arm pre-test/post-test prospective design	Creativity, Optimism, Planning, and Expert Information for Chemotherapy-Induced Peripheral Neuropathy (COPE-CIPN) Patient completes entire 50-min programme. Four modules of general CIPN and COPE information, neuropathic pain, upper-extremity neuropathy and lower-extremity neuropathy.	During treatment or immediately after infusion	14 participants with unspecified diagnosis, mainly Caucasians (92.86%) who received unspecified neurotoxic drugs	**Primary outcomes:** Usability Post-study System Usability Questionnaire (PSSUQ) Acceptability Acceptability E-scale Neuropathy symptoms and interference with daily activities Chemotherapy-Induced Peripheral Neuropathy Assessment Tool
Knoerl *et al.* (2018a) [11] USA	Single-arm pre-test/post-test prospective design	Carevive® Care Planning System (CPS) At each clinic visit, system prompts patient to rate intensity of CIPN symptom and interference of symptoms on daily activities. CPS platform generates symptoms summary page and creates a care plan that may be edited. Clinician reviews and edits the care plan before being sent to the patient.	During treatment (94.7%); treatment planning stage (5.3%)	75 female participants with breast cancer, mainly Caucasians (88%) and received docetaxel or paclitaxel	**Primary Outcome:** Patient Activation Patient Activation Measure **Secondary outcomes:** Feasibility, Stability, Acceptability/Satisfaction Clinicians = percentage of patients who received a care plan and average times providers reviewed care plans Patients = System Usability Scale, Adapted Acceptability E-scale with five extra questions Both = face-to-face informal feedback
Knoerl *et al.* (2018b) [53] USA	Multicentre, pilot, randomised, wait-list controlled trial	**I**: Usual care from primary provider + Proactive Self-Management Program for Effects of Cancer Treatment (PROSPECT) Participants completes a link ‘Steps For Me’. Website recommends modules based on patient’s responses. Patient may use modules as much as they desired; no additional encouragement to access modules were made. **C**: Usual care from primary provider; received access to intervention after completion of study-related surveys	After treatment (CIPN pain that persisted 3 months or longer after end of treatment)	60 male and female participants diagnosed with breast, gastrointestinal and other cancers, mainly Caucasians (91%), and received platinums or taxanes	**Primary outcome:** CIPN pain 7-day worst pain diary over 8 weeks **Secondary outcomes:** CIPN symptom severity EORTC Quality of Life Questionnaire (QLQ)-CIPN20 Average pain Numerical Rating Scale Pain interference Patient-Reported Outcomes Acceptability and Satisfaction Adapted Acceptability E-scale, semi-structured telephone descriptive interviews (positive and negative aspects of the intervention and barriers to access and use)
Kolb *et al.* (2018) [54] USA	Randomised controlled trial (sub-analysis)	**I:** SymptomCare@Home (SCH) to report symptoms and receive automated self-care coaching Patient calls automated phone system daily during chemotherapy treatment to prospectively report symptoms. Automated self-care coaching provided based on the specific symptom and severity. Automated system alerts nurse practitioner about poorly controlled symptoms who calls the patient to provide follow-up care based on a decision support system. **C:** Reported symptoms via SCH and told to contact their oncology team for any concerns	During treatment	252 participants diagnosed with various tumours (mainly breast cancer 45%), Caucasians (78.9%) and received either platinums, taxanes or combination platinums only	**Primary outcome:** Number of days at each symptom severity level symptom severity 1–10 scale (10 being the worst) **Secondary outcomes:** Distress associated with numbness1–10 numerical scale Interference with daily activities 1–10 numerical scale Helpfulness of self-care strategies 1–10 numerical scale Associated symptoms and utilisation of other services recorded Quality of Life (QoL) SF-36 Questionnaire Number of calls: recorded
Knoerl *et al.* (2019) [13] USA	Randomised controlled trial (sub-analysis)	**I:** Electronic Symptom Assessment-Cancer (ESRA-C) Patient self-report symptoms and quality of life measures at each visit. Programme may be accessed at home at their discretion. Moderate to severe symptom grading prompts patient to read self-care messages about the symptom, management and communicating with clinicians. Patient can track their symptoms at home. Clinicians receive a graphical representation of symptom severity. **C:** Limited access to the intervention; symptom-recording via ESRA-C; did not receive self-care messages about problematic symptoms and unable to track symptoms	Before treatment, during treatment and 2–4 weeks after treatment completion	220 participants diagnosed with various cancers, mainly Caucasians (87%) and received either taxanes, platinums or both	**Primary outcome:** Physical Function EORTC QLQ C-30 Physical Functioning Scale **Secondary Outcomes:** CIPN symptoms and QoL QLQ CIPN20 Perception of mood Patient Health Questionnaire 9 Pain intensity 0–10 pain intensity numerical scale
Exercise interventions					
Wonders et al. (2013) [55] USA	Single-arm pre-test/post-test prospective design	Structured, 10-week moderate intensity, home-based exercise programme	During and after chemotherapy treatment	Six female participants with breast cancer, mainly Caucasians (83%) who received taxanes or vinorelbine.	**Primary outcomes:** Pain Assessment Leeds Assessment of Neuropathic Symptoms and Signs questionnaire QOL McGill QoL questionnaire **Secondary Outcome:** Adherence numerical data
Streckman et al. (2014) [56] Germany	Randomised controlled trial	**I:** Supervised, bi-weekly, 36-week aerobic endurance, sensorimotor and strength training **C:** Received standard care including physiotherapy.	During treatment	61 patients diagnosed with lymphoma who received unspecified neurotoxic drugs. Ethnicity was not reported.	**Primary outcome:** Quality of life EORTC QLQ-C30 questionnaire **Secondary outcomes:** Physical activity measurements numerical scales • Peripheral deep sensitivity (CIPN symptoms) • Activity levels • Balance control (cumulative sway paths, static and dynamic surface) • Incremental step test Side effects Subjective Global Assessment questionnaire (SGA) Level of anxiety and depression Hospital Anxiety and Depression Scale Cognitive impairment ‘Fragebogen Erlebter Defizite der Aufmerksamkeit’
Tofthagen et al. (2014) [50] USA	Single-arm pre-test/post-test prospective design	12-week, bi-weekly, 60-minute group strength and balance exercise programme	After treatment	Three patients with colorectal cancer who were treated with oxaliplatin and all Caucasians.	**Primary outcomes:** Balance, Strength and Gait • timed up and go (TUG) • unipedal stance time (UST) • Dynamic Gait Index (DGI) • modified Clinical Test for Sensory Interaction in Balance (mCTSIB) • Isokinetic dynamometry CIPN chemotherapy induced peripheral assessment tool (CIPNAT), Total Neuropathy Score (TNS) **Secondary Outcome:** Acceptability descriptive responses
Fernandes and Kumar (2016) [57] India	Single-arm pre-test/post-test prospective design	15-session lower limb closed kinematic chain balance exercises over 3 weeks	No data on stage of treatment	25 patients. No data about tumour group, drugs received or ethnicity	**Primary outcomes:** CIPN symptoms Total Neuropathy Score Balance Berg Balance Scale
Schwenk et al. (2016) [58] USA	Randomised controlled trial	**I:** 4-week, bi-weekly, 45-min interactive sensor-based balanced training **C:** CG continued their normal activity but did not receive any formal exercise programme at the site	After treatment	22 participants diagnosed with various haematological and solid cancers. Participant ethnicity and chemotherapy drugs were not reported.	**Primary outcome:** Balance Balansens ^TM^ sensors to measure sways **Secondary outcomes:** Severity of CIPN VPT score CIPN-related pain Numeric Rating Scale (NRS) score 0–10 Neuropathy-related numbness NRS score 0–10 Health-related QoL Short-Form Health Survey (SF-12) Fear of falling Falls Efficacy Scale-International (FES-I)
Kleckner et al. (2018) [59] USA	Randomised controlled trial (sub-analysis)	**I:** 6-week individualised, moderate-intensity, home-based progressive walking and resistance exercise programme **C:** Standard care for chemotherapy	During treatment	355 participants mainly diagnosed with breast cancer who received either taxanes or platinums; mainly Caucasians (85%).	**Primary outcome:** CIPN symptoms 0–10 scale, where 0= not present and10=as bad as you can imagine, during the last 7 days for: (1) numbness and tingling and (2) hot/ coldness in hands/feet **Secondary outcomes:** Adherence steps from a pedometer, minutes of resistance exercise, and RPE where 1= no exertion and 10=maximal exertion. Acceptability qualitative feedback survey.
Vollmers et al. (2018) [60] Germany	Randomised controlled trial	**I:** Individualised, bi-weekly sensorimotor, home-based exercise during and after chemotherapy treatment **C:** Received an instruction sheet informing them about the current state of science concerning physical activity in malignant diseases and suggesting a regular physical activity designed autonomously by the patients.	During treatment	36 female participants with breast cancer who received taxanes. Ethnicity was not reported.	**Primary outcome:** Balance Fullerton Advanced Balance Scale Upper and lower extremity strength hand dynamometry and chair rising test **Secondary outcomes:** Quality of Life European Organization on Research and Treatment of Cancer (EORTC), the QLQ-C30 (for all malignant diseases) and BR23 (for breast cancer), CIPN CIPN20 Fatigue Multidimensional Fatigue Inventory (MFI-20)
Zimmer et al (2018) [61] Germany	Randomised controlled trial	**I:** 8-week, bi-weekly, multimodal balance and strength exercise **C:** Received written standard recommendations to obtain physical fitness	During treatment	30 participants diagnosed with colorectal cancer treated with unspecified chemotherapy and/or targeted therapy. Ethnicity was not reported.	**Primary outcome:** CIPN FACT/GOG-NTX questionnaire **Secondary outcomes:** Endurance capacity 6MWT Strength h1RM Balance GGT-Reha (balance test)
McCrary et al. (2019) [62] Australia	Single-arm pre-test/post-test prospective design	Individualised, 8-week, 1-h, tri-weekly resistance, balance and cardiovascular exercise intervention	After treatment	29 participants with different cancer diagnoses, mainly breast (37.9%) and colorectal (27.6%) cancer. Most participants received taxanes or platinums or combination of these. Ethnicity was not reported.	**Primary outcomes:** CIPN Symptoms Total Neuropathy Score–clinical version (TNSc) and EORTC CIPN-20 questionnaire **Secondary outcomes:** Functional assessment tools • Mobility: 6-min walk test • Standing balance (Postural sway) • Lower limb strength and dynamic balance Disability CIPN Rasch Built Overall Disability Score (CIPN-R-ODS) Quality of life SF-36 Instrument Neurophysiology nerve conduction studies
Kneis et al. (2019) [63] Germany	Randomised controlled trial	**I:** Individualised, 12-week, bi-weekly, one-on-one balance and endurance training **C:** endurance training (twice weekly over 12 weeks)	After treatment	37 participants with colorectal cancer who received unspecified neurotoxic drugs. Ethnicity was not reported.	**Primary outcome:** Balance all the measurements were performed on a force plate **Secondary outcomes:** Self-reported CIPN symptoms and QoL EORTC QLQ-CIPN20 and EORTC QLQ-C30-questionnaire Cardiorespiratory fitness peak oxygen consumption (V ˙O2peak; mL·min^−1^·kg^−1^), maximum power output (Pmax_CPET; W/kg) and performance at the IAT (W/kg) measured during the maximum cardiopulmonary exercise test (CPET). Vibration sense first metacarpophalangeal joint, knuckle and patella via Rydel-Seiffer tuning fork with a graduating scale from 0 (no sensitivity) to 8 (highest sensitivity); repeated twice
Bland et al. (2019) [64] Canada	Randomised controlled trial	Supervised aerobic, resistance and balance training 3 days a week for 8–12 weeks. **I:** immediate exercise regimen during taxane chemotherapy **C:** delayed exercise after chemotherapy	**I:** during chemotherapy **C:** after chemotherapy	31 female participants with breast cancer, predominantly Caucasians (67%) who received taxanes.	**Primary outcome:** Patient-reported CIPN symptoms EORTC QLQ CIPN20 **Secondary outcomes:** QoL EORTC QLQ-C30 Quantitative Sensory Testing manual tuning fork, Neuropen peripheral neuropathy screening device and Neurotip Chemotherapy completion rate extracted from patient medical records, including reason for the dose adjustment.
Hammond et al. (2020) [65] Canada	Single-blind exploratory randomised controlled trial	**I:** 5–10 min nerve gliding exercise three times daily and education on how to manage symptoms of neuropathic pain, safety and protection. **C:** Standard care	From start to after treatment	48 female participants with breast cancer who were treated with taxanes. Ethnicity was not reported.	**Primary outcomes:** Neuropathic Symptoms and Signs Leeds Assessment for Neuropathic Symptoms (S-LANSS) Pain numeric pain rating scale (0–10) Disability of Arm, Shoulder and Hand DASH questionnaire **Secondary outcomes:** Grip strength hand dynamometry Quantitative Sensory Testing vibration analysis, pressure algometry Dual Nerve Disorder Neurosensory Analyser
Bahar-Ozdemir et al. (2020) [66] Turkey	Comparative quasi-experimental design	**I:** 20-min muscle strengthening and balance exercises, 5 days a week for 10 weeks. **C:** Standard care	During chemotherapy	60 participants who were diagnosed with various cancer tumours who were treated with taxanes, or platinums or both. Ethnicity was not reported.	**Primary outcomes:** Balance functional balance evaluation (Berg Balance Scale) and quantitative balance evaluation (NeuroCom Balance Master device) Neuropathic Pain painDETECT questionnaire (PDQ) QoL**:** EORTC QLQ C30

#### Behavioural studies

All BI studies originated from United States of America (USA) and participants (*n*=795) were recruited from outpatient chemotherapy units in various cancer centres. The six studies evaluated seven BIs. One study had a sample size of less than 20 [52]; all other studies had treatment group sample sizes between 30 and 100. Four BIs with 515 participants were delivered during chemotherapy treatment [11, 51, 52, 54]. One BI with 60 participants was delivered after treatment [53] and only one BI with 220 participants was delivered before, during and up to 2–4 weeks after treatment [13]. Only one study focussed on one CIPN symptom, i.e. pain [53]. Three studies were sub-analyses of interventions for multiple chemotherapy side effects including CIPN [13, 51, 54]; one study compared two behavioural interventions for breast cancer treatment symptoms including CIPN [51]. Four BI studies were RCTs, one was a single-arm pre-test/post-test prospective design and one was a single-arm post-test retrospective design. The outcomes measured and reported in the studies include severity of CIPN symptoms and neuropathic pain, impact on quality of life (QoL) and physical function, patient activation and intervention-specific outcomes. Three studies used validated patient-reported outcome measures (PROMs) for measuring CIPN outcomes such as the CIPN Assessment Tool [52] and QLQ-CIPN20 [13, 53]. One study used a physician-graded CIPN scoring scale [51], while another study used both a validated PROM for CIPN and an unvalidated PROM for interference of CIPN on daily activities scoring scale [54]. A validated Patient Activation Measure was used in one study to appraise the patient’s ability to actively manage their own health and symptoms [11].

#### Exercise studies

Studies involving exercise interventions were conducted in USA (*n*=4), Germany (*n*=4), Canada (*n*=2), Turkey (*n*=1), Australia (*n*=1) and India (*n*=1). Four EI studies used a single-arm pre-test/post-test prospective design, with study sample sizes ranging from three to twenty-nine. Out of eight RCTs, one was a sub-analysis of a primary RCT [59]. Control and intervention groups in all RCTs were similar in size but small in numbers (range: 11–19 participants per arm), except for one study with 170 in the intervention group and 185 in the control group [59]. One study adapted a quasi-experimental design whereby two groups received the intervention but at different stages of their chemotherapy treatment [66]. The main author in one study provided additional information about intervention delivery following email contact [57]. Outcomes measured were severity of CIPN symptoms and neuropathic pain, impact on quality of life (QoL) and physical function and intervention-specific outcomes. One study used a physician grading score, i.e. Total Neuropathy Score to measure CIPN symptoms [57] and one study used peripheral deep sensitivity testing only [56]. Five studies utilised validated tools such as the Leeds Assessment of Neuropathic Symptoms and Signs questionnaire [55], CIPN symptoms numeric rating scale [59], QLQ-CIPN20 [60], FACT-GOG NTx [61] and Pain DETECT questionnaire [66]. A combination of PROMs and physician grading score was used in one study [50]. The majority of the more recent studies used a combination of validated PROMs and Quantitative Sensory Testing [58, 62–65].

### Intervention synthesis: behavioural interventions

**Fig. 2:**
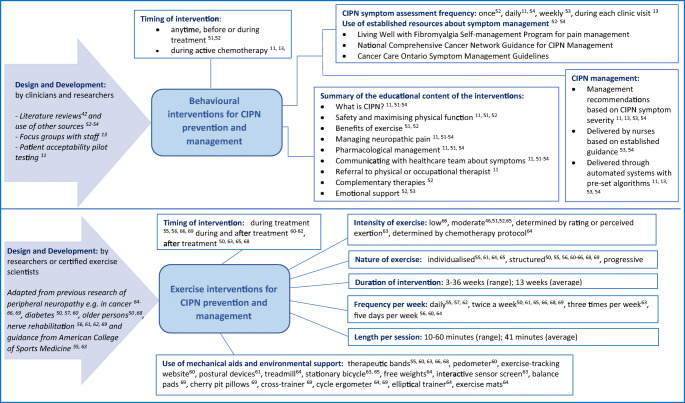
Representation of characteristics of behavioural and exercise interventions for chemotherapy-induced peripheral neuropathy

Figure 2 presents the characteristics of behavioural and exercise interventions identified in this review. The six BI studies included in the review generated seven behavioural interventions, two of which were compared in one study [51].

#### Scope and mechanisms of action

Most interventions did not solely focus on CIPN management but also included management of other chemotherapy side effects. Interventions included self-reporting of CIPN symptoms and neuropathic pain which were assessed alongside other cancer and treatment side effects [13, 51, 53, 54]. All interventions contained educational components about CIPN and management. Some interventions referred participants to established sites with information about CIPN and its management through local links and national cancer organisations [51, 53, 54]. Key topics found across interventions included general information about CIPN, safety and physical function, how to report symptoms, pharmacological and non-pharmacological interventions and referral to therapists.

Explicit details that explained mechanisms of action and how they caused a change of behaviour or achieved outcomes were lacking. Based on descriptions provided in the studies, we identified the possible mechanisms of action of the interventions listed below:

- provides regular patient reminders to monitor and report their symptoms [13, 51]
- encourages discussing symptom management with their nurse about managing their symptoms [51]
- assists patients to acquire information, if needed [51]
- gives automated advice for symptom management [51]
- increases patient’s knowledge about CIPN, safety and management [11, 13, 52, 54]
- assists patients to be able to self-manage symptoms [11, 52, 53]
- encourages patient-provider decision-making [11]
- increases patient activation to manage own symptoms [11]
- provides access to symptom management strategies to use at their own pace [13, 53]
- allows patients to report their symptoms [54]
- provides coaching on safety-related self-care [54]
- alerts nurse practitioner to poorly controlled symptoms [54]
- provides instructions to enable patients to communicate CIPN symptoms to clinicians [54]

#### Underpinning conceptual models

**Fig. 3:**
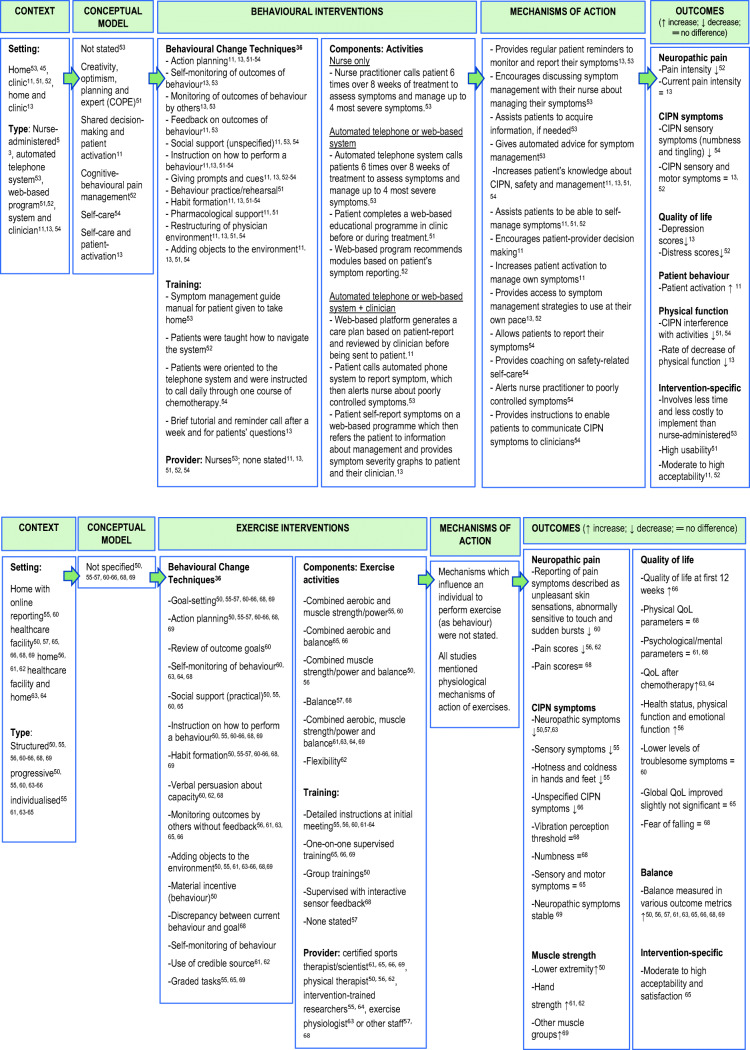
Summary of current evidence—behavioural and exercise interventions for chemotherapy-induced peripheral neuropathy

No study explicitly applied a conceptual model for intervention development. Some of the included studies used behavioural or psychological concepts to describe the rationale or goal of the elements essential to the interventions. These include creativity, optimism, planning and expert [52], shared decision-making [11], patient activation [11, 13], cognitive-behavioural pain management [53] and self-care [13, 54]. The level of reporting was insufficient to enable identification of conceptual models and theoretical bases of interventions. The application of BCT Taxonomy v.1 [36] identified a total of 12 behaviour change techniques (BCTs) used in the intervention arms as illustrated in Fig. 3. The BCTs identified as present in all interventions were action planning, instruction on how to perform a behaviour and habit formation. Giving prompts and cues were used in all but one intervention [52].

Conceptual constructs from studies and mechanisms of action, based on our interpretation, are presented in Fig. 3.

#### Components and context

##### Components (activities)

In two interventions, no further follow-up was provided after the initial activity which involved completing a 50-min programme of four informational modules [52] and a cross-sectional pain symptom assessment that generated recommended educational modules for participants to use as they wish [54]. Five interventions assessed self-reported severity of CIPN symptoms during chemotherapy treatment [11, 13, 51, 53, 54]. Self-reported severity assessments were conducted once [53], daily [11, 54], weekly [51] or during each clinic visit [13]. Of these five studies, two provided telephone coaching on CIPN management when CIPN symptoms were severe [51, 54]. When symptoms were poorly controlled, the system alerted the nurse practitioner to make telephone contact with the participant to provide follow-up care [54]. In one intervention, when a patient reported CIPN above the severity threshold, a nurse provided CIPN management coaching [51]. On the other hand, one intervention generated a care plan based on CIPN severity which the clinician could edit or tailor before sending to the patient by [11]. In one intervention, moderate to severe CIPN symptom grading prompted participants to read self-care messages on the website [13]. Interventions which recommended management strategies based on severity of symptoms were facilitated through guidelines provided to nurses and patients [51] and pre-set algorithms embedded in automated systems [11, 13, 51, 54].

##### Setting and type

One intervention was delivered entirely by a nurse [51] to patients in their home setting, one by nurses via telephone [54] and one through an automated phone system [51]. Two interventions were delivered via a web-based format accessed by participants in clinics [52] or at home [53]. Two web-based interventions also included clinician interaction, who were mainly the doctors who saw patients in clinics [11, 13].

##### Training

Several interventions included patient training on how to use or navigate a web-based system [13, 53] and a telephone system [54]. Participants in two interventions were given a symptom management guide booklet to take home with them [51].

#### Intervention development

Three studies did not describe the process and who was involved in intervention design and development [51, 53, 54]. A common feature among these three studies was the use of already-established resources that were available to patients or staff. In one of the studies, extensive literature reviews on strategies for managing CIPN symptoms at home and guidance for nursing management of CIPN were conducted by researcher clinicians to develop the intervention [52]. One study involved members of the multidisciplinary team consisting of oncology physicians, nurses, social worker and scientists in developing the interventions. They provided recommendations for managing CIPN symptoms and participated in the iterative process of prototype development through focus groups [13, 67]. Only one study obtained feedback from patients through a pilot study [11, 68]. However, no studies included patients and/or staff who deliver the intervention during the intervention design and development process.

#### Outcomes

Table 2 summarises the effects of BIs on neuropathic pain, CIPN symptoms, QoL, patient activation and physical function, and should be read in conjunction with Table 1.

**Table 2:**
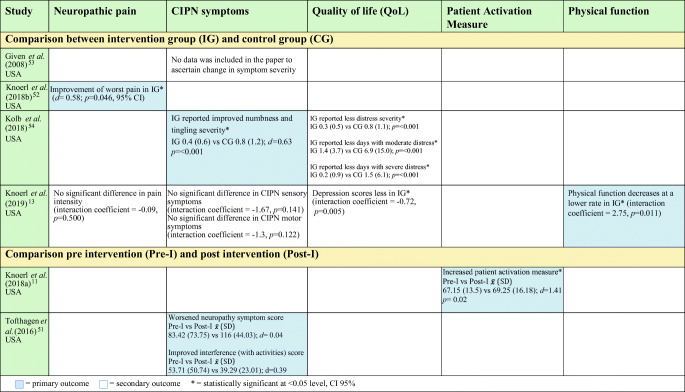
Effect of behavioural interventions on outcomes

As primary outcome, one BI study showed improvement of worst pain with large effect size in the intervention group (IG) (*d*= .58; *p*=0.046, 95% CI) [53]. The intervention (PROSPECT website) included cognitive-behavioural pain management strategies and information to assist participants manage pain and other cancer treatment side effects. Content were presented in written and video formats and may be accessed as often as possible by patients. Patients were given training at baseline on site navigation and on how to complete a questionnaire webpage which then recommended relevant content based on patient responses. No additional encouragement was provided by staff to access the platform [53].

However, in another study which investigated ESRA-C, a web-based electronic care planning intervention that allowed symptom self-reporting and provided self-care education, no significant statistical difference was observed between the IG and CG for current pain intensity as secondary outcome (*d*= − 0.09, *p*= 0.500, 95% CI) [13]. The ESRA-C intervention provided patients with self-care education and guidance on how to communicate CIPN symptoms to their clinicians. It also allowed clinicians to monitor symptom progression over time based on PROMs which also generated summary reports, e.g. graphs and journal. ESRA-C IG depression scores were significantly lower (*p=* 0.005) and physical function decreases at a lower rate when compared with the CG [13].

CIPN sensory symptoms was a primary outcome for one BI study [54] which showed statistically significant improvement of symptoms in favour of the intervention (*d*=− 0.63, *p*=0.001, 95% CI). The intervention, SymptomCare@Home, is a telephone-based symptom-monitoring and self-care coaching system. It alerted the nurse practitioner when CIPN symptoms exceed a preset threshold, who then provided telephone-based follow-up care based on guidelines [54]. SymptomCare@Home IG also reported significantly less days of moderate (*p=* <0.001) or severe (*p=* 0.001) distress relative to CIPN symptoms [54].

BI studies that explored CIPN sensory and motor symptoms as secondary outcomes after receiving PROSPECT [53] and ESRA-C [13] interventions did not show statistically significant difference between the control and intervention groups.

Patient activation level was the primary outcome of one BI study [11] which showed improvement (*d*=1.41, *p*= 0.02) from baseline ($\overline{x}$= 67.15; SD= 13.5) to post-intervention delivery ($\overline{x}$= 69.25; SD= 16.18). The intervention, Carevive ® care planning system (CPS), is an electronic platform that collects patient-reported data of symptoms including severity of sensory, motor and autonomic symptoms, and functional interference. Based on responses, the platform generated a summary page that highlighted symptom severity scores and key issues for their next clinic visit. CPS then created a care plan which were emailed to patients. The care plan contained links regarding CIPN treatment options, which the clinician edited to individualise according to patient’s needs and to add referrals. It also encouraged patients to discuss their CIPN symptoms with their clinicians [11].

#### Adherence

Few studies reported feasibility or acceptability. Adherence was not measured but participants identified issues they experienced when using interventions such as distractions and interruptions [52], lack of time [11, 52, 53], lack of information about non-painful neuropathy [11], recommendations were not found useful [52], navigational difficulties and small font sizes [52], difficulty logging in due to lost password or software issues [53] and complex interface [13].

### Intervention synthesis: exercise interventions

#### Mechanisms of action

All studies mentioned physiological mechanisms of action of the exercise components; however, no studies mentioned psychological mechanisms that may influence an individual to perform exercise.

#### Underpinning conceptual models

No study reported conceptual models on which an intervention was based. We used the behaviour change taxonomy [37] to identify and evaluate behaviour change techniques contained in exercise interventions based on intervention descriptions in the published papers. All studies contained goal setting (behaviour), action planning and habit formation. Instruction on how to perform a behaviour was present in all interventions except for one, which may be likely due to poor reporting of the intervention [57]. These five most common BCTs were present in the only methodologically strong study reviewed [59].

#### Components and context

##### Components (activities) and timing

Four interventions were delivered after chemotherapy treatment [50, 58, 62, 63]. Six interventions were delivered while participants were on active chemotherapy treatment (55–57, 64, 66, 69] while two interventions extended exercise activities for a few more weeks after treatment [60, 65]. One intervention was delivered to either participants who were undergoing chemotherapy or those who had completed treatment [55]. The exercise interventions ranged from 3 to 36 weeks in duration, with activities performed daily and two, three or five times a week. Most interventions were performed twice a week with each session ranging from 10 to 60 min. Not all studies clearly reported the intensity of exercise interventions. One intervention was low-intensity [56] and four were moderate-intensity [46, 52, 53, 63] but how intensity was measured was not explained in the studies. In one study, the rating of perceived exertion by participants determined the exercise intensity [62]; another depended on chemotherapy treatment protocol periods wherein low-intensity exercises, rather than aerobic-intensity exercises, were prescribed when chemotherapy side effects were expected to happen [64]. Intervention activities comprised either as a single type of exercise or combination of aerobic (improves cardiorespiratory fitness), muscle strength/power (increasing skeletal muscle strength, power, endurance and mass), balance (improve ability to withstand challenges from postural sway or destabilisation) and flexibility exercise (increases joint range of motion) [69]. Only one study involved purely hand exercises [65]. Mechanical aids were provided depending on the nature of prescribed activity such as therapeutic bands [55, 56, 58, 59, 62], pedometer [55], exercise-tracking website [55], posture devices [60], treadmill [64], stationary bicycle [62, 63], free weights [64], interactive sensor screen [62], balance pads [61], cherry pit pillows [61], cross-trainer [61], cycle ergometer [61, 64], elliptical trainer [64] and exercise mats [64].

##### Training

Instructions about the intervention were provided to patients at the start of training by clinical research associates who received training [59], a sports scientist or physical/physiotherapist [60, 64–66], a person described as supervisor [58] and unspecified personnel [55]. Following provision of instruction during the first training, one study used interactive sensor feedback technology to guide participants while exercising [58].

##### Setting and type

Five interventions were completely home-based (55, 56, 60–62], one was a combination of performing physical activities at home and at a healthcare facility [62, 64], and all other interventions were conducted solely in a healthcare facility. All exercises were structured but some were individualised [59, 60, 62–64] and progressive in terms of difficulty level [50, 55, 56, 59, 62–64].

#### Intervention development

Illustrated in Fig. 2, all EIs were developed using existing evidence from the literature and expert guidelines. Guidance was adapted from exercise interventions used for individuals experiencing other forms of neuropathy, cancer and older persons with balance problems. Two interventions were underpinned by guidelines designed by the American College of Sports Medicine [59, 62]. Patients or participants were not consulted about the various components of the intervention or involved in the design and development of these.

#### Outcomes

Table 3 shows a summary of effects of EIs on neuropathic pain, CIPN symptoms, QoL, balance and muscle strength. This should be read in conjunction with Table 1.

**Table 3:**
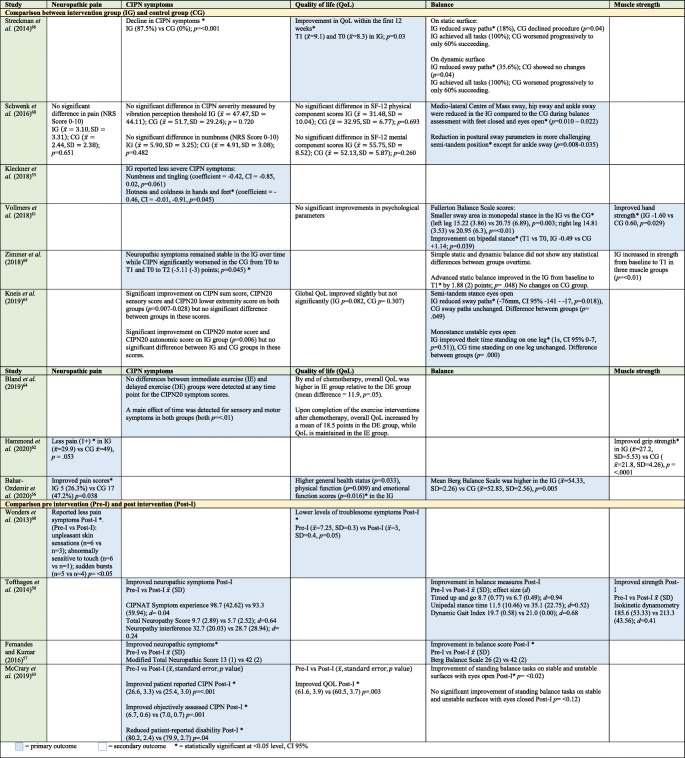
Effect of exercise interventions on outcomes

No significant difference in pain scores (*p*=0.651) between the IG and the CG was noted on a study where pain was a secondary outcome [58]. However, statistically significant lower pain scores between groups [66], less reported pain symptoms between groups (*p* = .053) [65] and less reported pain symptoms within groups (*p*= <0.05) [55] were observed in studies where pain was the primary outcome.

In three single-arm pre-post test studies, there were significant improvements of CIPN symptoms after the exercise interventions [50, 57, 62]. Mixed results were observed in RCTs; two studies showed no significant difference in CIPN scores between IG and CG [58, 63]. On the other hand, significant decline in CIPN symptoms was observed in IG but not in CG in two of the exercise studies [56, 59]. CIPN symptoms in IG were stable while CIPN significantly worsened in the CG from baseline to follow-up visits (*p*=0.045) [61]. A study which compared delayed exercise (DE) and immediate exercise (IE) showed no difference in CIPN symptom scores at any timepoint [64]. Interestingly, at the end of their chemotherapy, overall QoL was significantly higher among patients in the IE group than in the DE group (mean difference = 11.9, *p*=.05) [64].

Significant intervention effects on QoL was observed in two pre-post test exercise studies [55, 62] and three RCTs [47, 57, 59]. In one RCT, there was improvement in QoL within the first 12 weeks (after 12 weeks: $\overline{x}$=9.1; baseline: $\overline{x}$=8.3; *p*=0.03) [56], while higher general health status (*p*=0.033), physical function (*p*=0.009) and emotional function scores (*p*=0.016) were observed in another RCT study [66]. Post-intervention, significantly lower levels of troublesome symptoms (pre: $\overline{x}$=7.25, SD=0.3 vs post: $\overline{x}$=3, SD=0.4, *p*=0.05) [55] and improved QoL were reported ($\mathrm{pre}:\overline{x}$= 61.6, SD=3.9 vs ($\mathrm{post}:\overline{x}$= 60.5, SD=3.7, *p*=.003) [62]. One RCT did not show any significant difference in physical QoL component scores between groups (*p*=0.693) [58], while two studies did not show any difference between IG and CG on mental (*p*=0.260) or psychological component scores [58, 60]. There was slight improvement in global QoL in both IG (*p*=0.082) and CG (*p*=0.307) in one study, but both were not significant [63].

Exercise interventions with physical activity components specifically for balance [50, 56–58, 60–63, 66] were evaluated for intervention effect on balance except for one study [65]. All six RCTs [56, 58, 60, 61, 63, 66] and three pre-post studies [50, 57, 62] showed statistically significant improvement of balance scores (*p*= 0.00–0.005). Due to heterogeneity of metrics to measure balance outcomes, intervention elements (e.g. activities, length and duration) and study designs, we were unable to pool the results. Four studies had significant positive effect on strength of hand grip [60, 65], lower extremities [50] and three muscle groups [61].

##### Adherence

No adverse events related to performing the exercises were reported. Reasons for non-adherence or non-completion of full intervention dose include:

- Lack of motivation to continue [55, 57]
- Too busy to meet exercise goals [55, 57, 63, 65]
- Balance issues unrelated to neuropathy [50]
- Perceived lack of benefit from intervention [50]
- Lack of transport to study centre [58]
- Medical event, disease progression or physical issues unrelated to study [58, 59, 61–63, 65, 66]
- Overwhelmed by the intervention [57]
- Psychological reasons [61]
- Consent withdrawn by participant [60, 62]
- Offered no reason or unspecific personal reasons [59, 60, 64]

Based on the review findings, we present a summary of the current evidence of behavioural and exercise interventions for prevention and management of CIPN (Fig. 3). This should be read in conjunction with Table 1 and Fig. 2.

## Discussion

Of the 1,954 papers identified, nineteen met all inclusion criteria. Reporting of study design and intervention details was mostly limited or incomplete. There was heterogeneity in study designs, outcome measures and the components of interventions. Most studies were appraised as moderate to strong in methodological design. However, greater rigour is needed in terms of study design and blinding those who were performing objective symptom assessments. Specifically, the person delivering the intervention should not be the same person measuring the effectiveness of that intervention [70].

Intervention studies included in this review were atheoretical or did not provide sufficient information to develop conclusions regarding the use of theory. Instead, conceptual psychological constructs were implied as underpinning the behavioural interventions; however, their use was rare and not explicitly stated. Interventions which were based on conceptual constructs focused mainly on patient’s behaviour with little or no consideration of the multi-faceted patient experience of CIPN [4]. To address complexity, interventions that comprise several interacting components and target various organisational levels are valuable [71, 72]. In addition, rationales provided for using exercise activities were centred on how these may improve balance, CIPN symptoms and physical functioning; the focus being on performing the exercise rather than changing psychological processes that can result in people performing the exercise.

Theoretical or conceptual frameworks can assist researchers in their understanding of which factors to target to improve the likelihood of achieving desired behaviours. The use of frameworks provides guidance on understanding the interaction and relationships between the content of the intervention, selection of BCTs, the process of implementation and the factors that influence implementation [29]. For instance, a cognitive-behavioural theory may be used as framework to develop a detailed understanding of patients’ perception of CIPN and their behavioural responses to potentially developing and experiencing CIPN symptoms. Evidence shows the benefit of theory-driven and evidence-based intervention designs on maximising effectiveness, sustainability and successful implementation of interventions [73, 74]. Further, theoretical or conceptual frameworks can aid in the formation of evidence-based intervention guidelines that can be used for future research. We recommend that researchers wishing to intervene, prevent or improve patient’s CIPN symptoms incorporate a theoretical or conceptual framework into their intervention plan.

The use of behaviour change techniques within the interventions was evident, ranging from four to nine BCTs across all interventions. Most of the interventions utilised five key behaviour change strategies, i.e. action planning, habit formation, instruction on how to perform a behaviour, providing prompts and cues and goal setting. Although these five were the most widely reported behaviour techniques, we do not know if they were associated with improving CIPN symptoms, neuropathic pain or quality of life due to lack of adequate information on their mechanisms of action to inform any conclusions.

Most behavioural interventions (BIs) provided support and advice on how to assess and manage CIPN symptoms each time participants used the interventions during their treatment period. These may be vital prompts for patients to recognise and report CIPN symptoms early, especially as the amount of information received by patients before chemotherapy tends to obscure relevant information about CIPN [4]. However, there is no adequate data to ascertain if and how these helped patients develop useful behavioural responses for coping with CIPN symptoms. In general, studies that evaluated the effect of the BI on CIPN symptoms showed promising results although the wide range of different treatment components and outcome measures make it impossible to determine which intervention is most effective. Except for one study [11], none of the studies evaluated the impact of their intervention on behavioural outcomes such as patient activation. This is surprising because mapping against the BCT Taxonomy [37] indicated they contained many behaviour change techniques. It was difficult, therefore, to identify active ingredients of intervention mechanisms to develop recommendations for further intervention modelling. Based on our interpretation, studies that were rated strongest methodologically [11, 13, 53] evaluated BIs that may create an effect on outcomes by increasing patient’s knowledge about CIPN, safety and management [11, 13]; assisting patients to be able to self-manage symptoms [11, 53]; and providing access to symptom management strategies to use at their own pace [13, 53]. One intervention also provided regular patient reminders to monitor and report their symptoms [13], and another encouraged patient-provider decision-making [11] as well as increased patient activation to manage own symptoms [11]. Positive outcomes of these BIs include improved pain [53] and lower depression scores [13], slower decline of physical function [13] and increased patient activation [11].

We also noted that most BIs were administered during chemotherapy treatment; none were provided to patients with lingering CIPN symptoms after treatment. This could negatively affect patient experience as there is some evidence that 30% of patients will have CIPN symptoms 6 months or more after finishing treatment [56]. This is concerning as it coincides with when patients are either being seen less in clinics or discharged from specialist oncology care and have limited access to support.

Similar to findings of earlier reviews on exercise interventions (EIs) for CIPN [10, 12], it is hard to evaluate the relative benefits of different EIs due to intervention variability in terms of types of activities, length, duration and structure of exercises and the wide range of objective and self-reported outcome measures used. This presents challenges when choosing which intervention should be recommended to patients, especially when none of the interventions were directly compared within a single study. To advance understanding of the benefits of exercise on CIPN outcomes, the research community ought to agree on intervention assessment and outcome measures. Only one study rated as methodologically strong showed promising EI outcomes in reducing CIPN symptoms through a 6-week structured, progressive and individualised home-based exercise intervention with combined aerobic and muscle strength exercises [59]. Interventions that assessed outcomes such as neuropathic pain, CIPN symptoms, quality of life, balance and muscle strength showed favourable results (although the extent of potential benefits was difficult to judge). A recent study, which was published after the systematic search date of this review, also suggests that muscle strength and balance exercises reduce CIPN symptoms and improve QoL [75].

Exercise interventions delivered during and after chemotherapy treatment were modelled on existing EIs used in other neuropathic conditions. This implies the use of exercise in CIPN management is nascent and requires further investigation. Only one study incorporated exercise and an educational package on how to manage CIPN symptoms [65]; only outcomes related to physiological effects of exercise were measured. We recognise the importance and benefits of the biological mechanisms underpinning exercise interventions. However, we are also interested in understanding the psychological mechanisms of action that may have influenced the individual to perform the exercise for managing CIPN.

There were intervention adherence issues in several exercise studies although these were not reported in detail. Other studies which explored exercise in cancer patients during and after treatment also reported low adherence [76, 77]. Behavioural and socio-demographic factors such as distance to facility, length of exercise activities, person’s willingness to change exercise behaviour and motivation to perform exercise were shown to affect adherence to exercise activities among cancer patients [78–82]. To enhance adherence, exercise activities should be near the patient’s home, encourages family involvement and includes feedback and coaching by trainers [83]. In this review, reasons for non-adherence in exercise interventions for CIPN included lack of motivation, becoming too overwhelmed and perceived lack of benefit; all of these could potentially be addressed through behavioural interventions. It may be worth investigating whether a combined behavioural and exercise intervention in CIPN is feasible and useful.

In all reviewed studies, intervention design and development processes were researcher-led and lacked involvement from the patients and/or clinical staff who deliver the intervention. Only one study consulted patients for feedback on a pre-developed intervention [11, 68]. Early involvement of those who will use and implement the intervention in the intervention design and development stages helps to identify contextual factors that might later inhibit or facilitate implementation [37]. More importantly, interventions should target the needs of the specific population for which they are intended. A recent review highlighted the need to conduct preliminary qualitative research to identify the issues of concern from the perspective of those experiencing them and/or those who will be delivering the intervention [84]. In addition, the use of methodological approaches, such as Experience-Based Co-Design, that engage both patients and staff in intervention design and development [85] has been shown to improve relevance, help understanding of implementation challenges and increase willingness and capacity to implement healthcare interventions by clinicians and service users [86–88]. Hence, we propose patient and clinical staff involvement for future development of interventions for preventing and managing CIPN. As mentioned earlier, future research should also seek to develop targeted behaviour change interventions, based on a sound theoretical underpinning.

Strengths of this review include a structured, systematic and broad search strategy. Even so, findings should be interpreted within the context of its limitations. The findings of the review were limited by the sample recruited. Most patients across studies were Caucasians reducing the generalisability of the findings to other ethnicities. Because studies were carried out mainly in the USA, Canada, Australia and Germany, care must be taken in generalising these findings to individuals from other countries owing to differences in health systems and cultural norms. Consideration of cultural norms is vital to improve the efficacy of behavioural physical activity interventions [89, 90]. To effect behaviour change, mode of intervention delivery should be guided by local context and what is acceptable to those who will access the intervention [91]. It is important to note that the review findings were based on limited intervention detail within published articles. We adopted approaches to make comparisons between interventions and to confirm interpretations. For example, we used a well-recognised approach developed by Michie et al. [37] to identify the behavioural change components within the interventions (which were not reported in the studies). In addition, emerging interpretations were shared with the co-authors for discussion and agreement throughout the review process. We also developed a summary of current interventions to illustrate our interpretation of the detailed components of the interventions, their assumed mechanisms of action and the associated outcomes. Due to the nature of the literature, we are unable to comment on prevention and management separately.

## Conclusion

This review identified and appraised evidence relating to existing behavioural and exercise interventions for preventing or managing symptoms of CIPN. The use of behaviour change techniques within the interventions was largely implicit. The lack of adequate information in the included studies prevents firm conclusions to be drawn on whether the most widely used behaviour techniques were effective. We are unable to recommend a specific behavioural intervention due to variability in the study design, outcomes and components of the behavioural interventions. Behavioural interventions that increase patient’s CIPN knowledge, improve their self-management capacity and provide access to symptom management at their own pace show potential benefits but more research is required. Similarly, the heterogeneity of the exercise interventions in terms of types of activities, length, duration and structure of exercises and the wide range of objective and self-reported outcome measures used makes it difficult to recommend a particular exercise intervention over others. But results of this review suggest potential benefits of exercise on intended outcomes. In all reviewed studies, intervention design and development processes were researcher-led and lacked involvement from the patients and/or clinical staff. We recommend that researchers wanting to develop interventions to prevent or improve patient’s CIPN symptoms should incorporate a clear theoretical or conceptual framework into their intervention plan and involve the specific patient population group and those who will deliver the intervention in the design and development process.

### Supplementary Information

ESM 1 (PDF 209 kb)
