# Supplementary material for: A systematic review of behavioural and exercise interventions for the prevention and management of chemotherapy-induced peripheral neuropathy symptoms
Source: J Cancer Surviv. 2021 Mar 12;17(1):254–77. doi: 10.1007/s11764-021-00997-w (PMC9971149; doi:10.1007/s11764-021-00997-w)
Supplement: Supplementary file 1 — (PDF 209 kb) [file 11764_2021_997_MOESM1_ESM.pdf]

A systematic review of behavioural and exercise interventions for the prevention and management of chemotherapy-induced peripheral neuropathy symptoms

Journal of Cancer Survivorship
Tanay MAL, Armes J, Moss-Morris R, Rafferty AM and Robert G
Corresponding author: Tanay MAL mary.tanay@kcl.ac.uk

Online material

Table S-1. Exemplar of the search protocol for Medline.

1. chemotherapy.mp.
2. peripheral neuropathy.mp.
3. neuropathy.mp.
4. 2 or 3
5. 1 and 4
6. intervention.mp.
7. program$.mp. or exp *Programs/
8. system.mp.
9. exercise.mp
10. exp *Physical Activity as Topic
11. follow-up.mp.
12. model.mp.
13. 6 or 7 or 8 or 9 or 10 or 11 or 12
14. psycholog*.mp.
15. exp Behavior Therapy/ or exp Behavior/ or behavio#r.mp.
16. education*.mp.
17. exp *Patient Education as Topic/ or psychoeducation*.mp.
18. self-management.mp. or exp Self Care/ or exp Self-Management/
19. motivational interview*.mp. or Motivational Interviewing /
20. 14 or 15 or 16 or 17 or 18 or 19
21. 5 and 13 and 20
22. limit 21 to (english language and yr="2000 -Current" and english and humans and cancer and "humans only (remove records about animals)")

Table S-2. Results of quality assessment of studies using the EPHPP a Quality Assessment Tool for Quantitative Studies (Thomas et al. 2004)

STUDIES INCLUDED	CRITERIA						
	**Selection bias**	**Study design**	**Confounders**	**Blinding Process**	**Data collection methods**	**Sample attrition**	**Overall**
**Behavioural intervention studies**							
Given et al. (2008)	Moderate	Strong	Moderate	Weak	Moderate	Moderate	Moderate
Tofthagen et al. (2016)	Moderate	Moderate	Weak	Moderate	Strong	Moderate	Moderate
Knoerl et al. (2018a)	Moderate	Moderate	Moderate	Moderate	Strong	Strong	Strong
Knoerl et al. (2018b)	Strong	Strong	Moderate	Strong	Strong	Moderate	Strong
Kolb et al. (2018)	Moderate	Strong	Weak	Moderate	Strong	Moderate	Moderate
Knoerl et al. (2019)	Moderate	Strong	Moderate	Moderate	Strong	Strong	Strong
**Exercise intervention studies**							
Wonders et al. (2013)	Moderate	Moderate	Moderate	Moderate	Weak	Weak	Weak
Streckman et al. (2014)	Strong	Moderate	Moderate	Weak	Moderate	Weak	Weak
Tofthagen et al. (2014)	Weak	Moderate	Weak	Weak	Moderate	Weak	Weak
Fernandes & Kumar (2016)	Weak	Moderate	Weak	Weak	Weak	Weak	Weak
Schwenk et al. (2016)	Moderate	Strong	Moderate	Moderate	Strong	Moderate	Moderate
Kleckner et al. (2018)	Moderate	Strong	Strong	Moderate	Strong	Strong	Strong
Vollmers et al. (2018)	Moderate	Weak	Moderate	Moderate	Strong	Moderate	Moderate
Zimmer et al (2018)	Moderate	Moderate	Moderate	Moderate	Strong	Strong	Moderate
McCrary et al. (2019)	Moderate	Moderate	Moderate	Moderate	Strong	Strong	Moderate
Kneis et al. (2019)	Strong	Moderate	Moderate	Moderate	Strong	Strong	Moderate
Bland et al. (2019)	Strong	Moderate	Strong	Weak	Moderate	Strong	Moderate
Hammond et al. (2020)	Moderate	Weak	Weak	Moderate	Weak	Weak	Weak
Bahar-Ozdemir et al. (2020)	Moderate	Moderate	Weak	Moderate	Moderate	Moderate	Moderate
